# Crystal structure of bis­{3-(benzo[*d*][1,3]dioxol-5-yl)-5-[6-(1*H*-pyrazol-1-yl)pyridin-2-yl]-4*H*-1,2,4-triazol-4-ido}nickel(II) methanol disolvate

**DOI:** 10.1107/S2056989025006851

**Published:** 2025-08-12

**Authors:** Kateryna Znovjyak, Sergiu Shova, Vazghen Nikolian, Andrii Khairulin, Igor O. Fritsky, Sergey O. Malinkin, Maksym Seredyuk

**Affiliations:** a Department of Chemistry, Taras Shevchenko National University of Kyiv, Volodymyrska Street 64, Kyiv, 01601, Ukraine; b Department of Inorganic Polymers "Petru Poni" Institute of Macromolecular Chemistry Romanian Academy of Science Aleea Grigore Ghica Voda 41-A Iasi 700487 Romania; c WimbleAI Inc., 548 Market Street, Unit 55559, San Francisco, California, USA; d ChemBioCenter Kyiv National Taras Shevchenko University Kyiv 02094 61 Winston Churchill Street Ukraine; Universität Greifswald, Germany

**Keywords:** crystal structure, nickel(II) complexes, neutral complexes, tridentate ligands

## Abstract

The unit cell of the title compound, [Ni(C_17_H_11_N_6_O_2_)_2_]·2CH_3_OH, consists of a neutral complex and two methanol mol­ecules. In the complex, the two tridentate 2-[3-(benzo[*d*][1,3]dioxol-5-yl)-1*H*-1,2,4-triazol-5-yl]-6-(1*H*-pyrazol-1-yl)pyridine ligands coordinate to the central Ni^II^ ion through nitro­gen atoms of the pyrazole, pyridine and triazole groups, forming a pseudo­octa­hedral coordination sphere. Neighbouring mol­ecules are linked through weak C—H(pz)⋯π(ph) inter­actions into monoperiodic chains, which are further linked through weak C–H⋯H/N/C inter­actions into diperiodic layers. The inter­molecular contacts were qu­anti­fied using Hirshfeld surface analysis and two-dimensional fingerprint plots, revealing the relative contributions of the contacts to the crystal packing to be H⋯H 38.4%, C⋯H/H⋯C 25.3%, N⋯H/H⋯N 14.1%, and O⋯H/H⋯O 11.8%. The average Ni—N bond distance is 2.085 Å. Energy framework analysis at the HF/3–21 G theory level was performed to qu­antify the inter­action energies in the crystal structure.

## Abstract 2

The neutral title compound bis­{3-(benzo[*d*][1,3]dioxol-5-yl)-5-[6-(1*H*-pyrazol-1-yl)pyridin-2-yl]-4*H*-1,2,4-triazol-4-ido}nickel(II) methanol disolvate has a distorted pseudo­octa­hedral coordination environment of the metal ion. As a result of their conical shape and polar nature, the mol­ecules stack in one-dimensional columns that are bound by weak hydrogen bonds into layers, which are arranged in three dimensions without inter­layer inter­actions below van der Waals radii.

## 1. Chemical context

A broad class of coordination compounds is represented by 3*d*-metal complexes based on tridentate bis­azole­pyridine ligands (Halcrow *et al.*, 2019 ▸; Suryadevara *et al.*, 2022 ▸), which find application in many fields, for example in catalysis (Xing *et al.*, 2014 ▸; Wei *et al.*, 2015 ▸) and mol­ecular magnetism (Suryadevara *et al.*, 2022 ▸). In the case of asymmetric ligand design, where one of the azole groups carries a hydrogen on a nitro­gen heteroatom and acts as a Brønsted acid, deprotonation can produce neutral complexes (Seredyuk *et al.*, 2014 ▸; Grunwald *et al.*, 2023 ▸). The periphery of the mol­ecule, *i.e.* ligand substituents, also plays an important role, determining the way the mol­ecules inter­act with each other, influencing the inter­molecular connectivity, inter­action energy and the organ­ization of the structure.
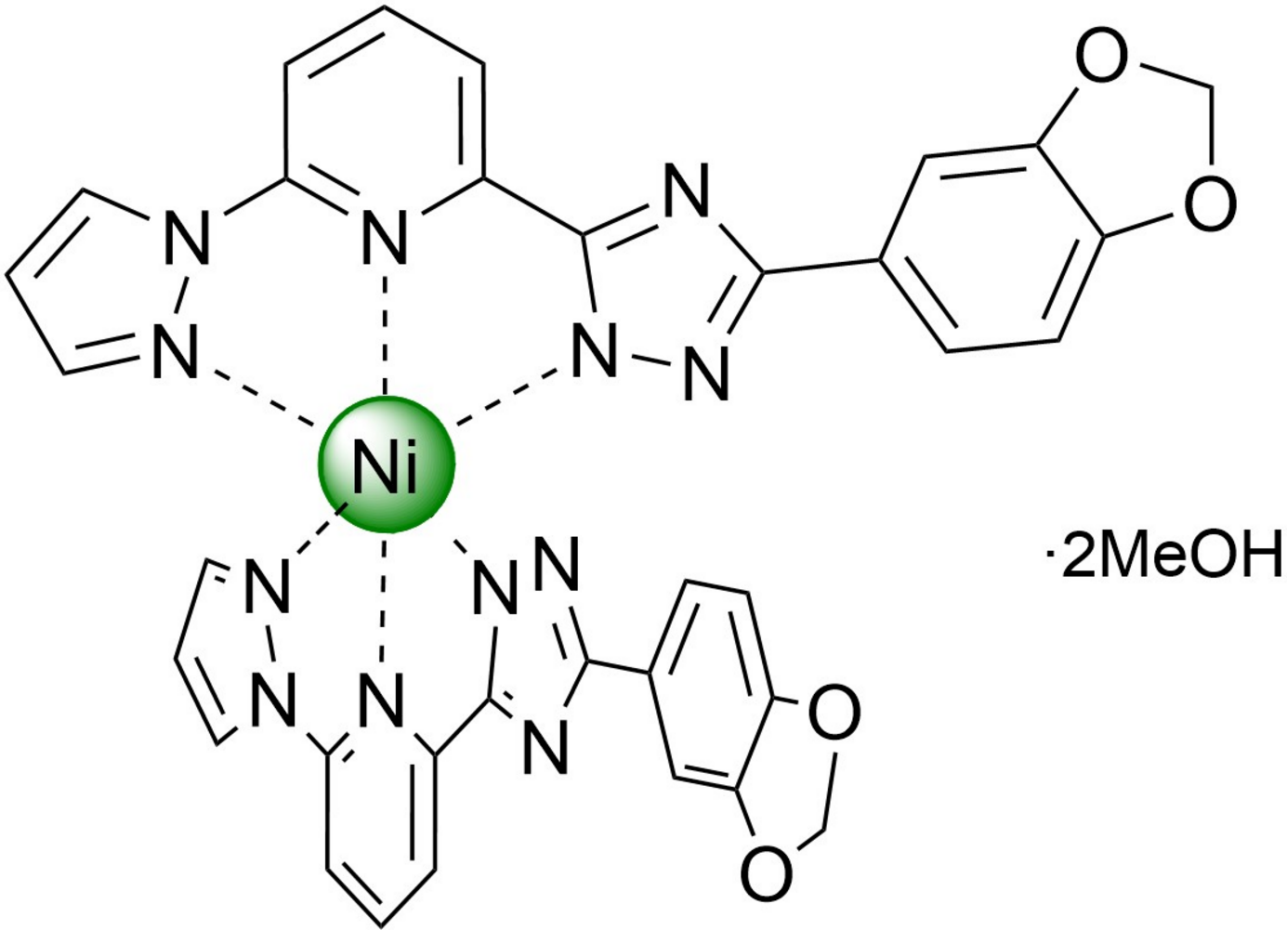


Encouraged by our results in spin-transition complexes of 3*d*-metals formed by N-heterocyclic ligands (Seredyuk *et al.*, 2006 ▸, 2007*a* ▸,*b* ▸, 2024*a* ▸; Piñeiro-López *et al.*, 2018 ▸), we report here a new neutral Ni^II^ complex based on the asymmetric deprotonated ligand 2-[3-(benzo[*d*][1,3]diox­ol-5-yl)-1*H*-1,2,4-triazol-5-yl)-6-(1*H*-pyrazol-1-yl]pyridine, which continues our lasting project on the study of 3*d*-metal complexes of bis­azole­pyridines and related organic polydentate ligands.

## 2. Structural commentary

**Figure 1:**
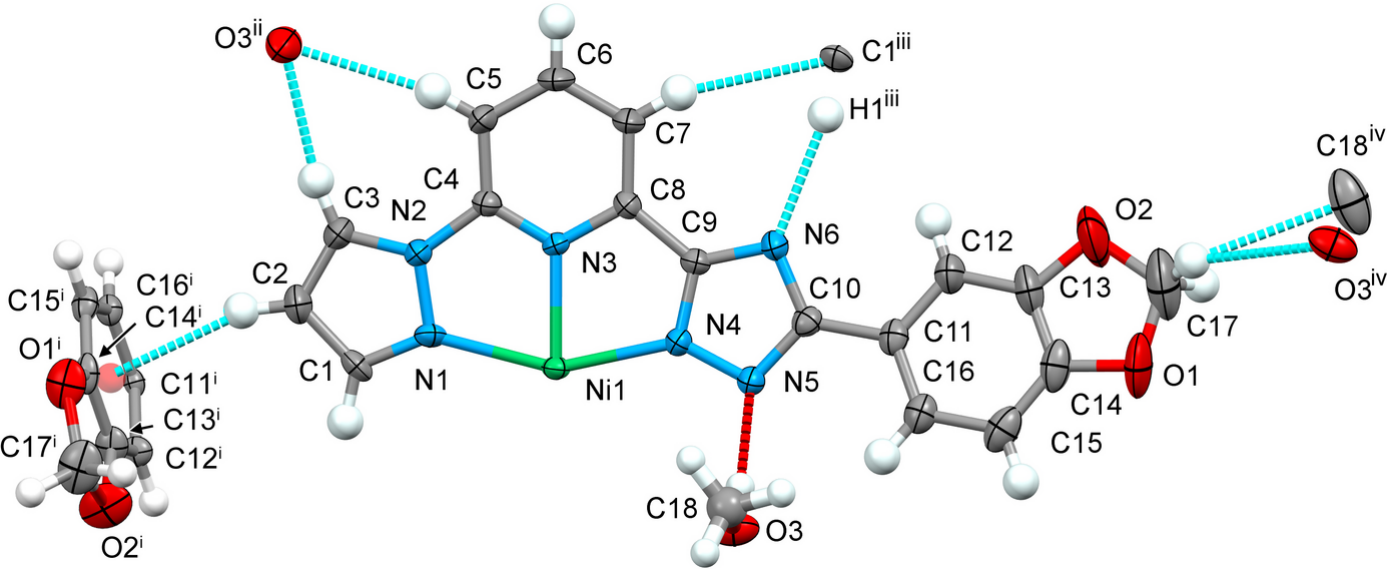
The mol­ecular structure in the asymmetric unit of the title compound and contact atoms with displacement ellipsoids drawn at the 50% probability level. The strong O—H⋯N (red) and weak C–H⋯N/C/O (cyan) hydrogen bonds are shown with the nearest neighbours. Symmetry codes: (i) 1 − *x*, 1 + *y*, 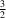 − *z*; (ii) −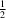 + *x*, 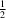 + *y*, 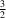 − *z*; (iii) 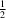 + *x*, −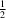 + *y*, 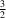 − *z*; (iv) −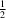 + *x*, 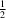 − *y*, 1 − *z*.

The complex has a conical structure with the nickel(II) residing on twofold rotation axis and half of the formula in the asymmetric unit. The phenyl ring of the benzodioxole moiety of the ligand is rotated by 18.6 (1)° relative to the almost planar pyrazole-pyridine-triazole (pz-py-trz) fragment. The independent methanol mol­ecule forms an O—H⋯N hydrogen bond with the trz ring of the ligand mol­ecule (Fig. 1 ▸). The central Ni ion of the complex has a distorted octa­hedral N_6_ coordination environment formed by the nitro­gen donor atoms of the two tridentate ligands. The average Ni—N bond length is 2.085 Å. Distortion indices were calculated to assess how much the coordination polyhedron deviates from ideal octa­hedral geometry. The average trigonal distortion parameters *Σ* = Σ_1_^12^(|90 − *φ*_i_|), where *φ*_i_ refers to the twelve *cis* angles N—Ni—N′ (Drew *et al.*, 1995 ▸), and *Θ* = Σ_1_^24^(|60 − *θ*_i_|), where *θ*_i_ is the angle generated by superposition of two opposite faces of an octa­hedron (Chang *et al.*, 1990 ▸) are 117.2 and 391.6°, respectively. The values reveal a deviation of the coordination environment from an ideal octa­hedron (where *Σ* = *Θ* = 0), which is, however, in the expected range for bis­azole­pyridine and similar ligands (see below). The calculated continuous shape measure [CShM(O_*h*_)] value relative to the ideal octa­hedral symmetry is 3.599 (Kershaw Cook *et al.*, 2015 ▸). The volume of the [NiN_6_] coordination polyhedron is 11.431 Å^3^.

## 3. Supra­molecular features

**Figure 2:**
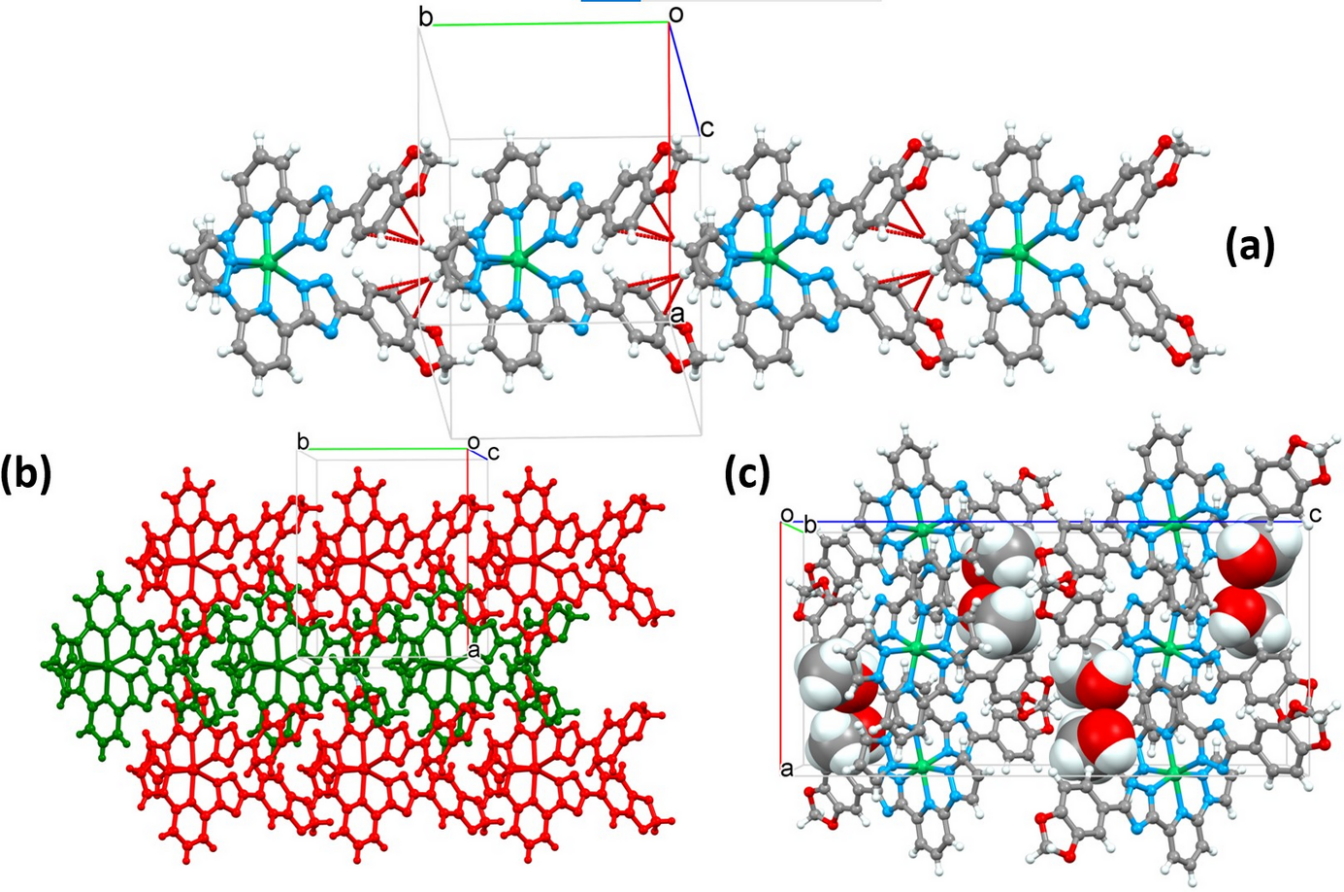
(*a*) A fragment of monoperiodic supra­molecular column formed by stacking of mol­ecules along the *b* axis; (*b*) supra­molecular diperiodic layers formed by stacking of the supra­molecular columns in the *ab* plane (for a better representation, each column has a different colour); (*c*) stacking of the diperiodic layers along the *b-*axis direction with the methanol mol­ecules in the voids.

Owing to the small head-group and large planar substituent at the tail, adjacent complex mol­ecules are inter­locked and inter­act *via* a weak, off-centre, almost perpendicular (83.6°) C—H(pz)⋯π(ph) inter­molecular contact between the pyrazole (pz) and phenyl (ph) groups with distances H2/C2⋯*C*_g_(ph) = 2.68/3.580 (4) Å. The formed monoperiodic supra­molecular chains extend along the *b-*axis direction with the stacking periodicity equal to 10.4956 (4) Å (= cell parameter *b*) (Fig. 2 ▸)*.* Through weak inter­molecular C—H(pz, py)⋯N/C inter­actions in the range 3.270 (4)–3.732 (5) Å (Table 1 ▸), neighbouring chains are joined into corrugated diperiodic layers in the *ab* plane. The layers stack without strong inter­layer inter­actions below the van der Waals radii; however, the solvent mol­ecules occupying voids between the layers participate in the bonding between separate layers. The methanol mol­ecule forms a strong O—H⋯N hydrogen bond with the deprotonated trz group and weak C—H⋯O hydrogen bonds with the CH_2_ group of the benzodioxole moiety belonging to a mol­ecule in a neighbouring chain. A list of the considered hydrogen-bonding inter­molecular inter­actions is provided in Table 1 ▸.

**Figure 3:**
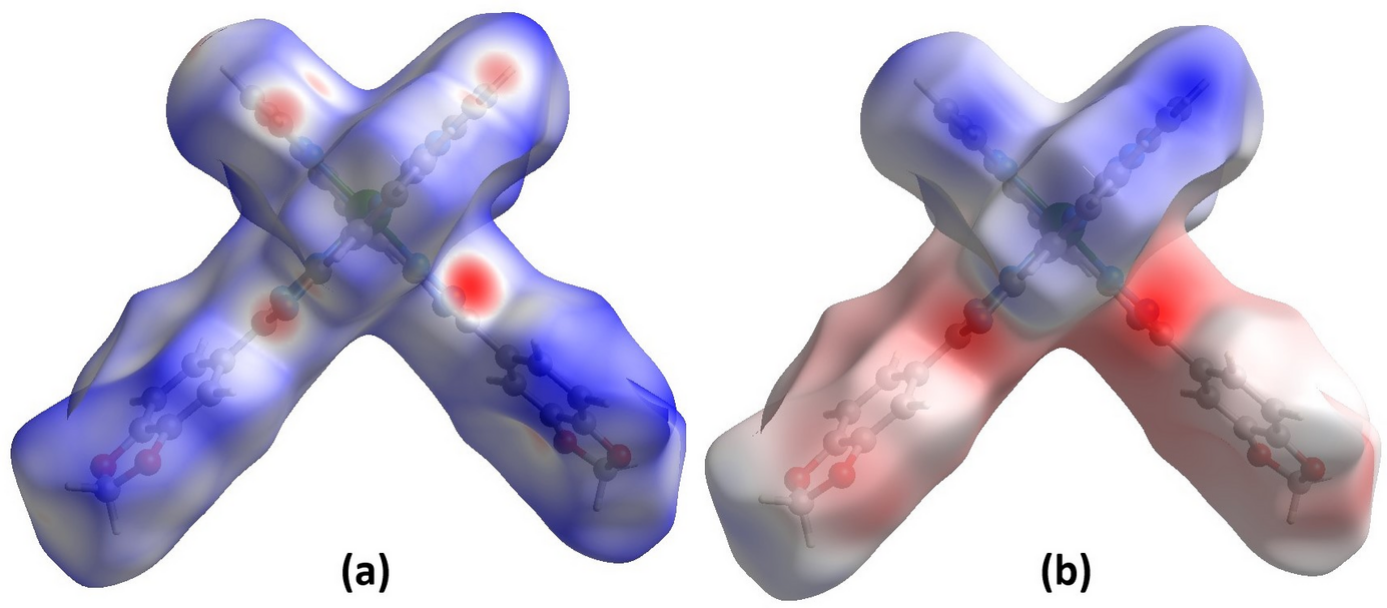
(*a*) A projection of *d*_norm_ mapped on the Hirshfeld surface identifying contact points or areas for inter­molecular inter­actions on the mol­ecule. Red/blue and white areas represent regions where contacts are shorter/larger than the sum and close to the sum of the van der Waals radii, respectively. (*b*) Electrostatic potential for the title compound mapped on the Hirshfeld surface. Red/blue and white areas represent regions where the charge is negative/positive or close to zero.

**Figure 4:**
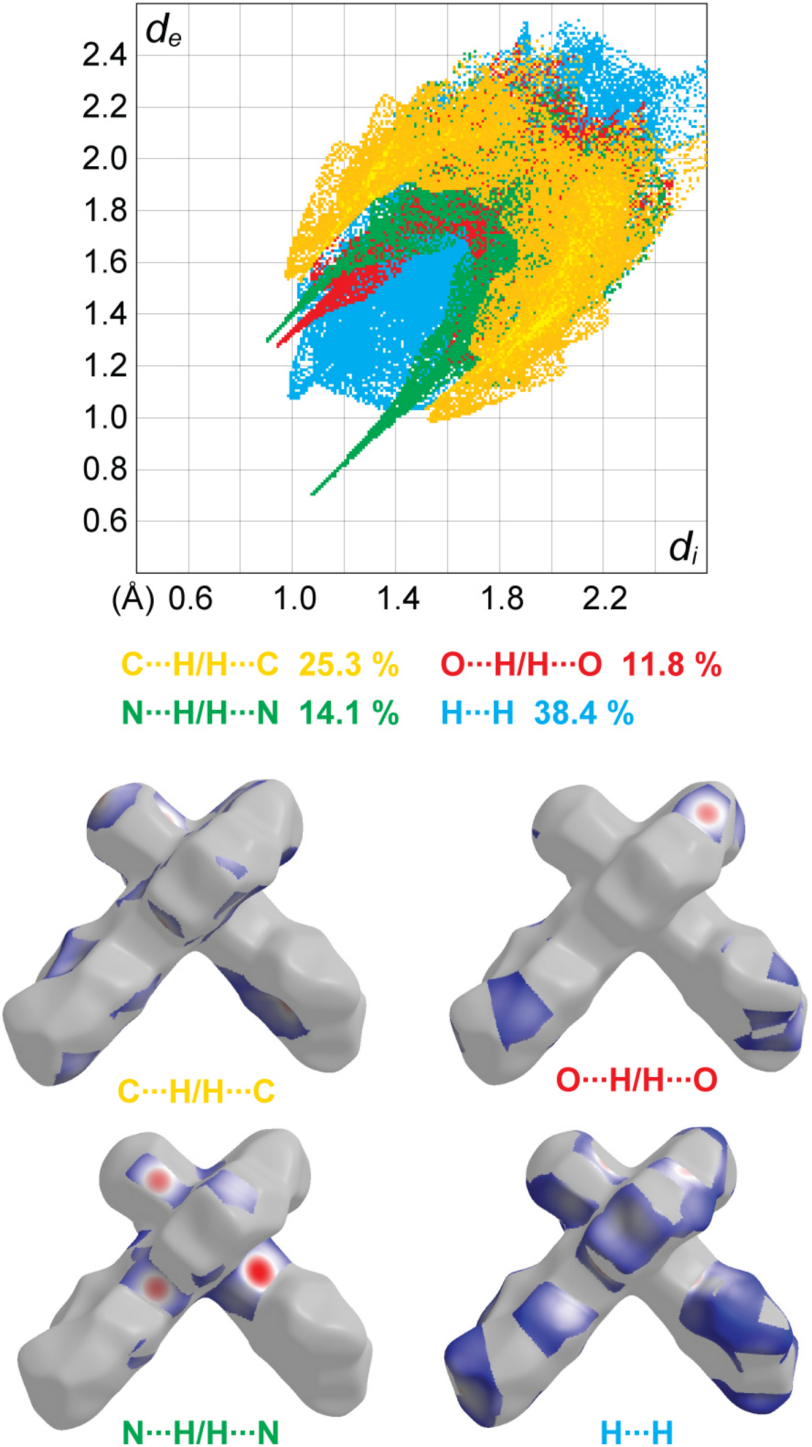
(*a*) Decomposition of the two-dimensional fingerprint plot into specific inter­actions. (*b*) A projection of *d*_norm_ mapped on the Hirshfeld surfaces, showing the specific inter­molecular inter­actions on the mol­ecule.

A Hirshfeld surface analysis was performed and the associated two-dimensional fingerprint plots were generated using *CrystalExplorer 21.5* (Spackman *et al.*, 2021 ▸), with a standard resolution of the three-dimensional *d*_norm_ surfaces (Fig. 3 ▸*a*). The pale-red spots symbolize short contacts and negative *d*_norm_ values on the surface corresponding to the inter­actions described above. The electrostatic potential energy calculated using the HF/3-21G basis set is mapped on the Hirshfeld surface (Fig. 3 ▸*b*). The negative charge localizes on the trz-ph moieties of the mol­ecules, while the pz-py moieties are relatively positively charged. The two-dimensional fingerprint plots, with their relative contributions to the Hirshfeld surface mapped over *d*_norm_, are shown for the H⋯H, C⋯H/H⋯C, N⋯H/H⋯N and O⋯H/H⋯O contacts in Fig. 4 ▸. At 38.4%, the largest contribution to the overall crystal packing is from H⋯H inter­actions, which are located in the middle region of the fingerprint plot. C⋯H/H⋯C contacts contribute 25.3%, and O⋯H/H⋯O 11.8%, resulting in pairs of characteristic wings. The N⋯H/H⋯N contacts, represented by a pair of sharp spikes in the fingerprint plot, make a 14.1% contribution to the surface.

**Figure 5:**
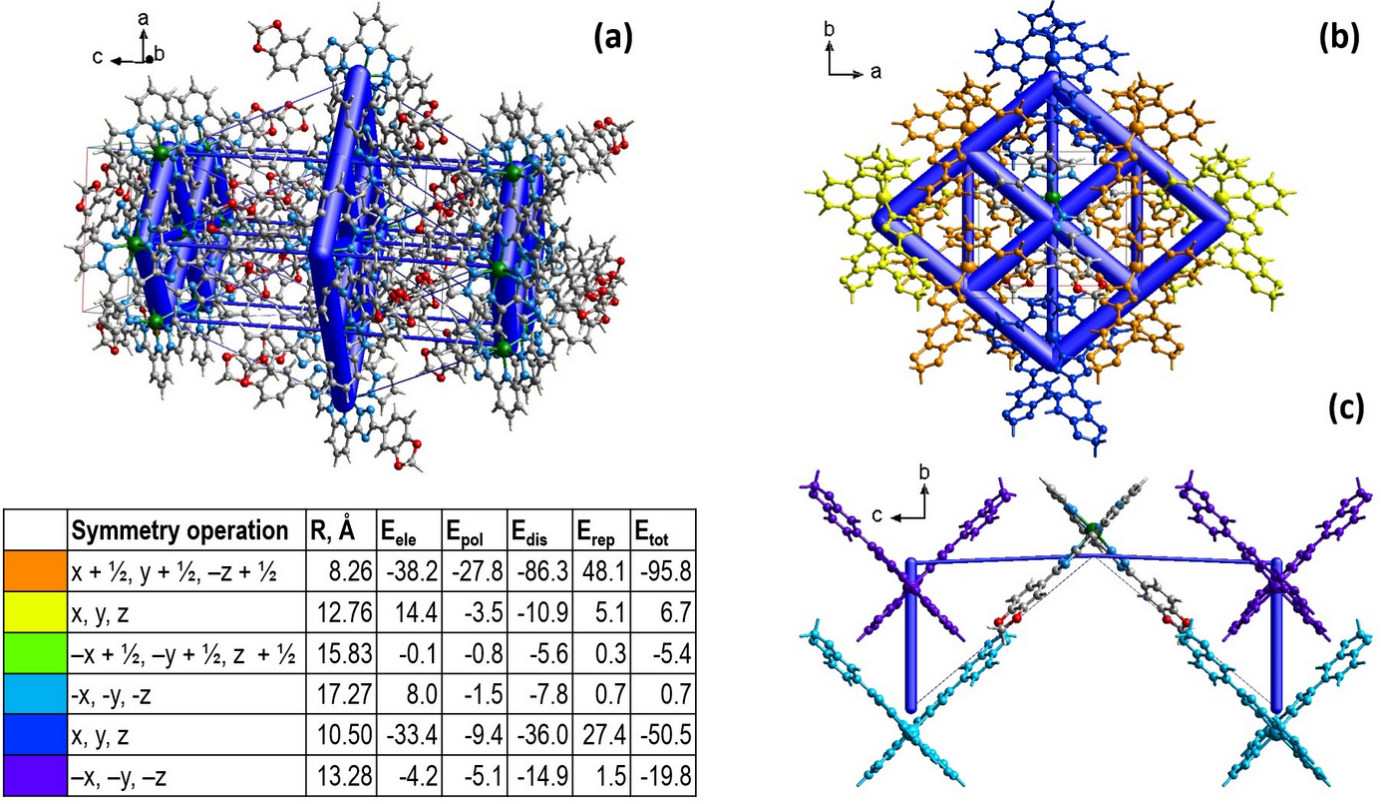
(*a*) The calculated energy frameworks, showing the total energy diagrams (*E*_tot_), (*b*) decomposition of the energy framework into the part corresponding to the inter­actions within a supra­molecular layer and (*c*) inter­layer inter­actions. In the table, the corresponding colour-coded energy values *E*_tot_ are provided, including their *E*_ele_, *E*_pol_, *E*_dis_, and *E*_rep_ components. Tube size is set at 100 scale.

The energy framework (Spackman *et al.*, 2021 ▸), calculated using the wave function at the HF/3-21G theory level, including the electrostatic (*E*_ele_), polarization (*E*_pol_), dispersion (*E*_dis_), repulsion (*E*_rep_) forces, and the total energy diagrams (*E*_tot_), is shown in Fig. 5 ▸. The cylindrical radii, adjusted to the same scale factor of 100, are proportional to the relative strength of the corresponding energies. The major contribution is due to dispersion forces (*E*_dis_), reflecting dominating inter­actions in the crystal of the neutral mol­ecules. The topology of the energy framework resembles the topology of the inter­actions within and between layers described above. The calculated value *E*_tot_ for the intra­chain inter­action is −50.5 kJ mol^−1^, and for inter­chain inter­actions is down to −95.8 kJ mol^−1^. The inter­layer inter­actions are represented by an energy of −19.8 kJ mol^−1^. The colour-coded inter­action mappings within a radius of 3.8 Å of a central reference mol­ecule together with full details of the various contributions to the total energy (*E*_ele_, *E*_pol_, *E*_dis_, *E*_rep_) are shown in the table in Fig. 5 ▸.

## 4. Database survey

A search of the Cambridge Structural Database (CSD, Version 5.42, last update August 2024; Groom *et al.*, 2016 ▸) reveals several similar neutral 3*d M*^II^ complexes with tridentate bis­azolpyridine ligands with a deprotonable azole groups, for example, of Ni^II^: YOCFAZ (Yuan *et al.*, 2014 ▸), ZOCKOT (Xing *et al.*, 2014 ▸), and ZOTVIP (Wei *et al.*, 2015 ▸); of Fe^II^: EGIDIL (Seredyuk *et al.*, 2024*b* ▸), LUTGEO (Senthil Kumar *et al.*, 2015 ▸), and XODCEB (Shiga *et al.*, 2019 ▸). In addition, there are related complexes based on phenanthroline-benzimidazole (DOMQUT; Seredyuk *et al.*, 2014 ▸), di­pyridyl­pyrrol (NIRLOT; Grunwald *et al.*, 2023 ▸). The values of the trigonal distortion and CShM(*O*_h_) change in correspondence to the length of *M*—N distances, and for shorter distances they are systematically lower than for the longer distances. Table 2 ▸ collates some key structural parameters of the complexes and of the title compound.

## 5. Synthesis and crystallization

The synthesis of the title compound is identical to that reported for a similar complex (Seredyuk *et al.*, 2022 ▸). It was produced by using a layering technique in a standard test tube. The layering sequence was as follows: the bottom layer contained a solution of [Ni(*L*_2_)](ClO_4_)_2_ prepared by dissolving *L* = 2-[3-(benzo[*d*][1,3]dioxol-5-yl)-1*H*-1,2,4-triazol-5-yl]-6-(1*H*-pyrazol-1-yl)pyridine (88 mg, 0.274 mmol) and Ni(ClO_4_)_2_·6H_2_O (50 mg, 0.137 mmol) in boiling acetone (5 ml), to which chloro­form (5 ml) was then added. The middle layer was a methanol–chloro­form mixture (1:10) (10 ml), which was covered by a layer of methanol (10 ml) to which 100 µl of NEt_3_ were added dropwise. The tube was sealed and violet plate-like single crystals appeared after 2 weeks (yield *ca*. 58%). Elemental analysis calculated for C_36_H_30_N_12_NiO_6_: C, 55.05; H, 3.85; N, 21.40. Found: C, 55.66; H, 3.48; N, 21.61.

## 6. Refinement details

Crystal data, data collection and structure refinement details are summarized in Table 3 ▸. The O-bound H atom was refined with *U*_iso_(H) = 1.5*U*_eq_(O); the hydrogen atom H3*A* was refined freely. All other H atoms were refined as riding [C—H = 0.95–0.99 Å with *U*_iso_(H) = 1.2–1.5*U*_eq_(C)]. An attempt to model a potential disorder in the oxalan moiety was unsuccessful as it did not improve the refinement. One reflection (002), which was obscured by the beamstop, was omitted as clear outlier.

## Supplementary Material

Crystal structure: contains datablock(s) I. DOI: 10.1107/S2056989025006851/yz2071sup1.cif

Structure factors: contains datablock(s) I. DOI: 10.1107/S2056989025006851/yz2071Isup2.hkl

Supporting information file. DOI: 10.1107/S2056989025006851/yz2071Isup3.cdx

CCDC reference: 2477384

Additional supporting information:  crystallographic information; 3D view; checkCIF report

## Figures and Tables

**Table 1:** Hydrogen-bond geometry (Å, °)

*D*—H⋯*A*	*D*—H	H⋯*A*	*D*⋯*A*	*D*—H⋯*A*
C3—H3⋯O3^i^	0.95	2.35	3.282 (5)	165
C5—H5⋯O3^i^	0.95	2.57	3.505 (4)	167
C7—H7⋯C1^ii^	0.95	2.68	3.605 (5)	163
C1—H1⋯N6^iii^	0.95	2.33	3.270 (4)	170
C17—H17*A*⋯C18^iv^	0.99	2.78	3.479 (7)	129
C17—H17*A*⋯O3^iv^	0.99	2.68	3.550 (6)	147
O3—H3*A*⋯N5	0.82 (4)	1.94 (4)	2.752 (4)	173 (4)

Symmetry codes: (i) 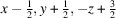; (ii) 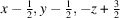; (iii) 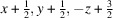; (iv) 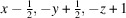.

**Table 2:** Computed distortion indices for the title compound and for similar complexes reported in the literature

CSD Refcode	Metal ion<	<*M*—N> (Å)	*Σ* (°)	*Θ* (°)	CShM(O_h_)
Title compound	Ni	2.085	117.2	391.6	3.60
YOCFAZ	Ni	2.088^*a*^	120.8^*a*^	397.6^*a*^	3.65^*a*^
ZOCKOT	Ni	2.086	121.0	375.9	3.78
ZOTVIP	Ni	2.110	124.9	382.4	3.55
EGIDIL	Fe	1.955	89.8	314.6	2.25
EGIDIL02	Fe	2.167	146.8	492.8	5.28
LUTGEO	Fe	1.933	85.0	309.6	2.10
XODCEB	Fe	1.950	87.4	276.6	1.93
DOMQUT	Fe	1.991	88.5	320.0	2.48
DOMQUT02	Fe	2.183	139.6	486.9	5.31
NIRLOT	Fe	1.939	77.3	255.6	1.68

Note: (*a*) average value.

**Table 3:** Experimental details

Crystal data	
Chemical formula	[Ni(C_17_H_11_N_6_O_2_)_2_]·2CH_4_O
*M*_r_	785.42
Crystal system, space group	Orthorhombic, *Pbcn*
Temperature (K)	200
*a*, *b*, *c* (Å)	12.7636 (4), 10.4956 (4), 26.5411 (12)
*V* (Å^3^)	3555.5 (2)
*Z*	4
Radiation type	Mo *K*α
μ (mm^−1^)	0.61
Crystal size (mm)	0.3 × 0.25 × 0.04

Data collection	
Diffractometer	Xcalibur, Eos
Absorption correction	Multi-scan (*CrysAlis PRO*; Rigaku OD, 2024 ▸)
*T*_min_, *T*_max_	0.982, 1.000
No. of measured, independent and observed [*I* > 2σ(*I*)] reflections	12665, 3146, 2236
*R*_int_	0.060
(sin θ/λ)_max_ (Å^−1^)	0.595

Refinement	
*R*[*F*^2^ > 2σ(*F*^2^)], *wR*(*F*^2^), *S*	0.053, 0.098, 1.04
No. of reflections	3146
No. of parameters	254
H-atom treatment	H atoms treated by a mixture of independent and constrained refinement
Δρ_max_, Δρ_min_ (e Å^−3^)	0.31, −0.35

Computer programs: *CrysAlis PRO* (Rigaku OD, 2024 ▸), *SHELXT* (Sheldrick, 2015*a* ▸), *SHELXL2018/3* (Sheldrick, 2015*b* ▸) and *OLEX2* (Dolomanov *et al.*, 2009 ▸).

## supplementary crystallographic information

### Bis{3-(benzo[d][1,3]dioxol-5-yl)-5-[6-(1H-pyrazol-1-yl)pyridin-2-yl]-4H-1,2,4-triazol-4-ido}nickel(II) methanol disolvate Crystal data

**Table:**

[Ni(C_17_H_11_N_6_O_2_)_2_]·2CH_4_O	*D*_x_ = 1.467 Mg m^−^^3^
*M**_r_* = 785.42	Mo *K*α radiation, λ = 0.71073 Å
Orthorhombic, *P**b**c**n*	Cell parameters from 2720 reflections
*a* = 12.7636 (4) Å	θ = 2.2–25.8°
*b* = 10.4956 (4) Å	µ = 0.61 mm^−^^1^
*c* = 26.5411 (12) Å	*T* = 200 K
*V* = 3555.5 (2) Å^3^	Plate, clear light violet
*Z* = 4	0.3 × 0.25 × 0.04 mm
*F*(000) = 1624	

### Bis{3-(benzo[d][1,3]dioxol-5-yl)-5-[6-(1H-pyrazol-1-yl)pyridin-2-yl]-4H-1,2,4-triazol-4-ido}nickel(II) methanol disolvate Data collection

**Table:**

Xcalibur, Eos diffractometer	3146 independent reflections
Radiation source: fine-focus sealed X-ray tube, Enhance (Mo) X-ray Source	2236 reflections with *I* > 2σ(*I*)
Graphite monochromator	*R*_int_ = 0.060
Detector resolution: 16.1593 pixels mm^-1^	θ_max_ = 25.0°, θ_min_ = 2.2°
ω scans	*h* = −10→15
Absorption correction: multi-scan (CrysAlisPro; Rigaku OD, 2024)	*k* = −12→9
*T*_min_ = 0.982, *T*_max_ = 1.000	*l* = −19→31
12665 measured reflections	

### Bis{3-(benzo[d][1,3]dioxol-5-yl)-5-[6-(1H-pyrazol-1-yl)pyridin-2-yl]-4H-1,2,4-triazol-4-ido}nickel(II) methanol disolvate Refinement

**Table:**

Refinement on *F*^2^	Primary atom site location: dual
Least-squares matrix: full	Hydrogen site location: mixed
*R*[*F*^2^ > 2σ(*F*^2^)] = 0.053	H atoms treated by a mixture of independent and constrained refinement
*wR*(*F*^2^) = 0.098	*w* = 1/[σ^2^(*F*_o_^2^) + (0.0254*P*)^2^ + 2.476*P*] where *P* = (*F*_o_^2^ + 2*F*_c_^2^)/3
*S* = 1.04	(Δ/σ)_max_ < 0.001
3146 reflections	Δρ_max_ = 0.31 e Å^−^^3^
254 parameters	Δρ_min_ = −0.35 e Å^−^^3^
0 restraints	

### Bis{3-(benzo[d][1,3]dioxol-5-yl)-5-[6-(1H-pyrazol-1-yl)pyridin-2-yl]-4H-1,2,4-triazol-4-ido}nickel(II) methanol disolvate Special details

**Table:**

Geometry. All esds (except the esd in the dihedral angle between two l.s. planes) are estimated using the full covariance matrix. The cell esds are taken into account individually in the estimation of esds in distances, angles and torsion angles; correlations between esds in cell parameters are only used when they are defined by crystal symmetry. An approximate (isotropic) treatment of cell esds is used for estimating esds involving l.s. planes.

### Bis{3-(benzo[d][1,3]dioxol-5-yl)-5-[6-(1H-pyrazol-1-yl)pyridin-2-yl]-4H-1,2,4-triazol-4-ido}nickel(II) methanol disolvate Fractional atomic coordinates and isotropic or equivalent isotropic displacement parameters (Å^2^)

**Table:**

	*x*	*y*	*z*	*U*_iso_*/*U*_eq_	
Ni1	0.500000	0.69588 (5)	0.750000	0.02073 (17)	
N3	0.34364 (17)	0.7067 (2)	0.76431 (9)	0.0210 (6)	
N6	0.29635 (19)	0.4568 (2)	0.67333 (11)	0.0294 (7)	
O3	0.6459 (2)	0.5655 (3)	0.61469 (11)	0.0479 (8)	
N4	0.43894 (17)	0.5611 (2)	0.70073 (10)	0.0235 (6)	
N2	0.38524 (18)	0.8644 (2)	0.81994 (10)	0.0268 (7)	
N1	0.48769 (18)	0.8386 (2)	0.80741 (10)	0.0246 (6)	
N5	0.47127 (18)	0.4777 (2)	0.66463 (10)	0.0262 (7)	
C4	0.3065 (2)	0.7917 (3)	0.79625 (12)	0.0251 (8)	
C10	0.3842 (2)	0.4172 (3)	0.64927 (13)	0.0263 (8)	
C7	0.1705 (2)	0.6386 (3)	0.74627 (15)	0.0388 (10)	
H7	0.123624	0.584348	0.728607	0.047*	
C9	0.3345 (2)	0.5459 (3)	0.70425 (13)	0.0245 (8)	
O2	0.2148 (3)	0.0879 (3)	0.54493 (14)	0.0921 (12)	
C6	0.1333 (2)	0.7279 (4)	0.77975 (15)	0.0431 (11)	
H6	0.059945	0.735043	0.785111	0.052*	
C5	0.2007 (2)	0.8075 (3)	0.80573 (13)	0.0363 (9)	
H5	0.175702	0.869689	0.828819	0.044*	
C12	0.2941 (3)	0.2482 (3)	0.60018 (15)	0.0412 (10)	
H12	0.234731	0.254816	0.621630	0.049*	
C11	0.3833 (2)	0.3232 (3)	0.60829 (13)	0.0290 (8)	
C8	0.2776 (2)	0.6292 (3)	0.73882 (12)	0.0250 (8)	
C3	0.3786 (3)	0.9550 (3)	0.85594 (14)	0.0380 (10)	
H3	0.316294	0.988162	0.870435	0.046*	
O1	0.3599 (3)	0.0627 (3)	0.49288 (12)	0.0741 (10)	
C16	0.4691 (3)	0.3094 (3)	0.57656 (13)	0.0363 (9)	
H16	0.529763	0.359884	0.582264	0.044*	
C14	0.3798 (3)	0.1522 (4)	0.52947 (15)	0.0462 (10)	
C15	0.4686 (3)	0.2229 (3)	0.53634 (15)	0.0452 (10)	
H15	0.527454	0.213721	0.514753	0.054*	
C2	0.4785 (3)	0.9895 (3)	0.86743 (15)	0.0406 (10)	
H2	0.499792	1.051567	0.891392	0.049*	
C13	0.2950 (3)	0.1662 (4)	0.56088 (16)	0.0485 (11)	
C1	0.5433 (2)	0.9155 (3)	0.83694 (13)	0.0287 (8)	
H1	0.617703	0.919390	0.837224	0.034*	
C18	0.6032 (4)	0.6536 (4)	0.58103 (17)	0.0741 (15)	
H18A	0.570036	0.608158	0.552967	0.111*	
H18B	0.658810	0.708858	0.568045	0.111*	
H18C	0.550579	0.705408	0.598456	0.111*	
C17	0.2564 (4)	0.0181 (5)	0.5035 (2)	0.0920 (18)	
H17A	0.211247	0.029769	0.473482	0.110*	
H17B	0.258379	−0.073876	0.511717	0.110*	
H3A	0.597 (3)	0.534 (4)	0.6305 (15)	0.048 (13)*	

### Bis{3-(benzo[d][1,3]dioxol-5-yl)-5-[6-(1H-pyrazol-1-yl)pyridin-2-yl]-4H-1,2,4-triazol-4-ido}nickel(II) methanol disolvate Atomic displacement parameters (Å^2^)

**Table:**

	*U*^11^	*U*^22^	*U*^33^	*U*^12^	*U*^13^	*U*^23^
Ni1	0.0147 (3)	0.0252 (3)	0.0223 (3)	0.000	0.0005 (3)	0.000
N3	0.0162 (13)	0.0235 (14)	0.0234 (17)	0.0020 (11)	0.0026 (11)	−0.0039 (14)
N6	0.0246 (15)	0.0327 (17)	0.0310 (18)	−0.0006 (12)	−0.0024 (13)	−0.0107 (15)
O3	0.0357 (16)	0.058 (2)	0.050 (2)	0.0056 (13)	0.0075 (14)	0.0173 (17)
N4	0.0220 (15)	0.0247 (15)	0.0237 (17)	0.0005 (11)	0.0009 (12)	−0.0042 (14)
N2	0.0208 (15)	0.0291 (16)	0.0305 (18)	0.0003 (11)	0.0014 (12)	−0.0093 (15)
N1	0.0169 (14)	0.0303 (15)	0.0265 (16)	−0.0007 (11)	0.0048 (12)	0.0012 (13)
N5	0.0231 (15)	0.0283 (15)	0.0271 (18)	0.0007 (11)	−0.0004 (12)	−0.0063 (15)
C4	0.0186 (17)	0.0298 (19)	0.027 (2)	0.0004 (14)	−0.0004 (14)	−0.0028 (18)
C10	0.0264 (18)	0.0256 (19)	0.027 (2)	0.0039 (14)	−0.0006 (15)	0.0003 (18)
C7	0.0173 (18)	0.049 (2)	0.051 (3)	−0.0011 (14)	0.0014 (17)	−0.020 (2)
C9	0.0190 (18)	0.0267 (19)	0.028 (2)	−0.0011 (13)	0.0006 (14)	−0.0016 (18)
O2	0.104 (3)	0.091 (3)	0.082 (3)	−0.043 (2)	−0.005 (2)	−0.047 (2)
C6	0.0161 (18)	0.058 (3)	0.056 (3)	0.0009 (16)	0.0055 (17)	−0.019 (2)
C5	0.0253 (19)	0.046 (2)	0.038 (2)	0.0009 (16)	0.0043 (16)	−0.019 (2)
C12	0.044 (2)	0.041 (2)	0.039 (3)	−0.0047 (17)	−0.0009 (18)	−0.013 (2)
C11	0.0362 (19)	0.0242 (19)	0.027 (2)	0.0044 (14)	−0.0051 (16)	−0.0002 (18)
C8	0.0214 (17)	0.0274 (18)	0.026 (2)	−0.0009 (13)	−0.0020 (14)	−0.0039 (17)
C3	0.030 (2)	0.039 (2)	0.045 (3)	0.0021 (16)	0.0047 (17)	−0.018 (2)
O1	0.122 (3)	0.054 (2)	0.047 (2)	−0.0098 (19)	−0.0071 (19)	−0.0288 (19)
C16	0.045 (2)	0.032 (2)	0.031 (2)	0.0050 (16)	−0.0009 (17)	−0.003 (2)
C14	0.079 (3)	0.032 (2)	0.027 (2)	0.005 (2)	−0.008 (2)	−0.003 (2)
C15	0.064 (3)	0.040 (2)	0.032 (2)	0.0120 (19)	0.0068 (19)	0.000 (2)
C2	0.041 (2)	0.039 (2)	0.042 (3)	−0.0059 (17)	−0.0033 (18)	−0.017 (2)
C13	0.066 (3)	0.036 (2)	0.044 (3)	−0.0125 (19)	−0.013 (2)	−0.011 (2)
C1	0.0235 (17)	0.0300 (19)	0.033 (2)	−0.0098 (14)	−0.0064 (16)	−0.0005 (19)
C18	0.113 (4)	0.058 (3)	0.051 (3)	0.032 (3)	0.026 (3)	0.011 (3)
C17	0.143 (5)	0.065 (4)	0.069 (4)	−0.031 (4)	−0.016 (4)	−0.026 (3)

### Bis{3-(benzo[d][1,3]dioxol-5-yl)-5-[6-(1H-pyrazol-1-yl)pyridin-2-yl]-4H-1,2,4-triazol-4-ido}nickel(II) methanol disolvate Geometric parameters (Å, º)

**Table:**

Ni1—N3^i^	2.035 (2)	O2—C17	1.425 (5)
Ni1—N3	2.035 (2)	C6—H6	0.9500
Ni1—N4^i^	2.078 (3)	C6—C5	1.383 (4)
Ni1—N4	2.078 (3)	C5—H5	0.9500
Ni1—N1	2.143 (3)	C12—H12	0.9500
Ni1—N1^i^	2.143 (3)	C12—C11	1.401 (4)
N3—C4	1.319 (4)	C12—C13	1.353 (5)
N3—C8	1.353 (4)	C11—C16	1.389 (4)
N6—C10	1.356 (4)	C3—H3	0.9500
N6—C9	1.336 (4)	C3—C2	1.360 (4)
O3—C18	1.396 (5)	O1—C14	1.375 (4)
O3—H3A	0.82 (3)	O1—C17	1.430 (5)
N4—N5	1.362 (3)	C16—H16	0.9500
N4—C9	1.345 (3)	C16—C15	1.401 (5)
N2—N1	1.376 (3)	C14—C15	1.366 (5)
N2—C4	1.410 (4)	C14—C13	1.374 (5)
N2—C3	1.350 (4)	C15—H15	0.9500
N1—C1	1.330 (4)	C2—H2	0.9500
N5—C10	1.343 (4)	C2—C1	1.394 (5)
C4—C5	1.383 (4)	C1—H1	0.9500
C10—C11	1.468 (4)	C18—H18A	0.9800
C7—H7	0.9500	C18—H18B	0.9800
C7—C6	1.376 (5)	C18—H18C	0.9800
C7—C8	1.385 (4)	C17—H17A	0.9900
C9—C8	1.461 (4)	C17—H17B	0.9900
O2—C13	1.379 (4)		
			
N3^i^—Ni1—N3	173.57 (14)	C4—C5—H5	121.8
N3—Ni1—N4	77.75 (10)	C6—C5—C4	116.3 (3)
N3—Ni1—N4^i^	106.77 (10)	C6—C5—H5	121.8
N3^i^—Ni1—N4	106.77 (9)	C11—C12—H12	121.0
N3^i^—Ni1—N4^i^	77.75 (9)	C13—C12—H12	121.0
N3—Ni1—N1	75.88 (9)	C13—C12—C11	118.0 (4)
N3—Ni1—N1^i^	99.53 (9)	C12—C11—C10	119.8 (3)
N3^i^—Ni1—N1^i^	75.88 (9)	C16—C11—C10	120.9 (3)
N3^i^—Ni1—N1	99.53 (9)	C16—C11—C12	119.3 (3)
N4—Ni1—N4^i^	94.21 (14)	N3—C8—C7	120.0 (3)
N4—Ni1—N1	153.63 (9)	N3—C8—C9	111.4 (3)
N4^i^—Ni1—N1^i^	153.63 (9)	C7—C8—C9	128.6 (3)
N4—Ni1—N1^i^	93.21 (10)	N2—C3—H3	126.6
N4^i^—Ni1—N1	93.21 (10)	N2—C3—C2	106.7 (3)
N1—Ni1—N1^i^	91.26 (14)	C2—C3—H3	126.6
C4—N3—Ni1	120.7 (2)	C14—O1—C17	104.8 (3)
C4—N3—C8	120.3 (3)	C11—C16—H16	119.2
C8—N3—Ni1	118.9 (2)	C11—C16—C15	121.7 (3)
C9—N6—C10	101.7 (2)	C15—C16—H16	119.2
C18—O3—H3A	107 (3)	C15—C14—O1	128.2 (4)
N5—N4—Ni1	140.06 (18)	C15—C14—C13	121.0 (4)
C9—N4—Ni1	114.1 (2)	C13—C14—O1	110.9 (4)
C9—N4—N5	105.8 (2)	C16—C15—H15	121.4
N1—N2—C4	117.6 (2)	C14—C15—C16	117.2 (4)
C3—N2—N1	111.6 (2)	C14—C15—H15	121.4
C3—N2—C4	130.7 (3)	C3—C2—H2	126.9
N2—N1—Ni1	112.28 (18)	C3—C2—C1	106.1 (3)
C1—N1—Ni1	143.5 (2)	C1—C2—H2	126.9
C1—N1—N2	104.2 (3)	C12—C13—O2	127.6 (4)
C10—N5—N4	105.5 (2)	C12—C13—C14	122.8 (4)
N3—C4—N2	113.3 (2)	C14—C13—O2	109.6 (4)
N3—C4—C5	123.3 (3)	N1—C1—C2	111.3 (3)
C5—C4—N2	123.3 (3)	N1—C1—H1	124.4
N6—C10—C11	123.3 (3)	C2—C1—H1	124.4
N5—C10—N6	113.3 (3)	O3—C18—H18A	109.5
N5—C10—C11	123.3 (3)	O3—C18—H18B	109.5
C6—C7—H7	120.6	O3—C18—H18C	109.5
C6—C7—C8	118.8 (3)	H18A—C18—H18B	109.5
C8—C7—H7	120.6	H18A—C18—H18C	109.5
N6—C9—N4	113.7 (3)	H18B—C18—H18C	109.5
N6—C9—C8	128.6 (3)	O2—C17—O1	109.1 (4)
N4—C9—C8	117.7 (3)	O2—C17—H17A	109.9
C13—O2—C17	105.5 (4)	O2—C17—H17B	109.9
C7—C6—H6	119.4	O1—C17—H17A	109.9
C7—C6—C5	121.2 (3)	O1—C17—H17B	109.9
C5—C6—H6	119.4	H17A—C17—H17B	108.3
			
Ni1—N3—C4—N2	−3.3 (4)	C10—C11—C16—C15	−178.1 (3)
Ni1—N3—C4—C5	176.9 (3)	C7—C6—C5—C4	−0.2 (6)
Ni1—N3—C8—C7	−177.2 (3)	C9—N6—C10—N5	0.3 (4)
Ni1—N3—C8—C9	0.9 (3)	C9—N6—C10—C11	−176.1 (3)
Ni1—N4—N5—C10	178.3 (3)	C9—N4—N5—C10	−0.6 (3)
Ni1—N4—C9—N6	−178.4 (2)	C6—C7—C8—N3	0.2 (5)
Ni1—N4—C9—C8	3.8 (4)	C6—C7—C8—C9	−177.5 (3)
Ni1—N1—C1—C2	179.5 (3)	C12—C11—C16—C15	0.4 (5)
N3—C4—C5—C6	0.3 (5)	C11—C12—C13—O2	−177.2 (4)
N6—C10—C11—C12	−15.0 (5)	C11—C12—C13—C14	1.1 (6)
N6—C10—C11—C16	163.5 (3)	C11—C16—C15—C14	0.0 (5)
N6—C9—C8—N3	179.5 (3)	C8—N3—C4—N2	179.7 (3)
N6—C9—C8—C7	−2.6 (6)	C8—N3—C4—C5	−0.1 (5)
N4—N5—C10—N6	0.2 (4)	C8—C7—C6—C5	0.0 (6)
N4—N5—C10—C11	176.5 (3)	C3—N2—N1—Ni1	−179.7 (2)
N4—C9—C8—N3	−3.1 (4)	C3—N2—N1—C1	0.3 (3)
N4—C9—C8—C7	174.8 (3)	C3—N2—C4—N3	−176.7 (3)
N2—N1—C1—C2	−0.4 (4)	C3—N2—C4—C5	3.1 (6)
N2—C4—C5—C6	−179.5 (3)	C3—C2—C1—N1	0.4 (4)
N2—C3—C2—C1	−0.2 (4)	O1—C14—C15—C16	179.4 (3)
N1—N2—C4—N3	−0.2 (4)	O1—C14—C13—O2	−1.6 (5)
N1—N2—C4—C5	179.6 (3)	O1—C14—C13—C12	179.9 (4)
N1—N2—C3—C2	−0.1 (4)	C14—O1—C17—O2	3.1 (5)
N5—N4—C9—N6	0.8 (4)	C15—C14—C13—O2	177.9 (4)
N5—N4—C9—C8	−176.9 (3)	C15—C14—C13—C12	−0.7 (6)
N5—C10—C11—C12	169.0 (3)	C13—O2—C17—O1	−4.1 (5)
N5—C10—C11—C16	−12.5 (5)	C13—C12—C11—C10	177.6 (3)
C4—N3—C8—C7	−0.1 (5)	C13—C12—C11—C16	−1.0 (5)
C4—N3—C8—C9	177.9 (3)	C13—C14—C15—C16	0.1 (6)
C4—N2—N1—Ni1	3.2 (3)	C17—O2—C13—C12	−178.1 (4)
C4—N2—N1—C1	−176.8 (3)	C17—O2—C13—C14	3.4 (5)
C4—N2—C3—C2	176.6 (3)	C17—O1—C14—C15	179.6 (4)
C10—N6—C9—N4	−0.7 (4)	C17—O1—C14—C13	−1.0 (5)
C10—N6—C9—C8	176.8 (3)		

Symmetry code: (i) −*x*+1, *y*, −*z*+3/2.

### Bis{3-(benzo[d][1,3]dioxol-5-yl)-5-[6-(1H-pyrazol-1-yl)pyridin-2-yl]-4H-1,2,4-triazol-4-ido}nickel(II) methanol disolvate Hydrogen-bond geometry (Å, º)

**Table:**

*D*—H···*A*	*D*—H	H···*A*	*D*···*A*	*D*—H···*A*
C3—H3···O3^ii^	0.95	2.35	3.282 (5)	165
C5—H5···O3^ii^	0.95	2.57	3.505 (4)	167
C7—H7···C1^iii^	0.95	2.68	3.605 (5)	163
C1—H1···N6^iv^	0.95	2.33	3.270 (4)	170
C17—H17*A*···C18^v^	0.99	2.78	3.479 (7)	129
C17—H17*A*···O3^v^	0.99	2.68	3.550 (6)	147
O3—H3*A*···N5	0.82 (4)	1.94 (4)	2.752 (4)	173 (4)

Symmetry codes: (ii) *x*−1/2, *y*+1/2, −*z*+3/2; (iii) *x*−1/2, *y*−1/2, −*z*+3/2; (iv) *x*+1/2, *y*+1/2, −*z*+3/2; (v) *x*−1/2, −*y*+1/2, −*z*+1.

### Hydrogen-bond geometry (Å, °).

**Table:**

*D–H···A*	*D–H*	*H···A*	*D···A*	*D–H···A*
C3–H···O3^ii^	0.95	2.36	3.282 (5)	165
C5–H···O3^ii^	0.95	2.57	3.505 (4)	167
C7–H···C1^iii^	0.95	2.68	3.605 (5)	163
N6···H–C1^iii^	0.95	2.33	3.270 (4)	170
C17–H···C18^iv^	0.99	2.78	3.479 (7)	129
C17–H···O3^iv^	0.99	2.68	3.550 (6)	147
O3–H···N6	0.82	1.94	2.753 (4)	172

Symmetry codes: (i) 1-x,1+y,1.5-z; (ii) -1/2+x,1/2+y,1.5-z; (iii) -1/2+x,-1/2+y,1.5-z; (iv) -1/2+x,1/2-y,1-z

## References

[bb1] Chang, H. R., McCusker, J. K., Toftlund, H., Wilson, S. R., Trautwein, A. X., Winkler, H. & Hendrickson, D. N. (1990). *J. Am. Chem. Soc.* **112**, 6814–6827.

[bb2] Dolomanov, O. V., Bourhis, L. J., Gildea, R. J., Howard, J. A. K. & Puschmann, H. (2009). *J. Appl. Cryst.* **42**, 339–341.

[bb3] Drew, M. G. B., Harding, C. J., McKee, V., Morgan, G. G. & Nelson, J. (1995). *J. Chem. Soc. Chem. Commun.* pp. 1035–1038.

[bb4] Groom, C. R., Bruno, I. J., Lightfoot, M. P. & Ward, S. C. (2016). *Acta Cryst.* B**72**, 171–179. 10.1107/S2052520616003954 PMC4822653 27048719

[bb5] Grunwald, J., Torres, J., Buchholz, A., Näther, C., Kämmerer, L., Gruber, M., Rohlf, S., Thakur, S., Wende, H., Plass, W., Kuch, W. & Tuczek, F. (2023). *Chem. Sci.* **14**, 7361–7380. 10.1039/d3sc00561e PMC10321519 37416721

[bb6] Halcrow, M. A., Capel Berdiell, I., Pask, C. M. & Kulmaczewski, R. (2019). *Inorg. Chem.* **58**, 9811–9821. 10.1021/acs.inorgchem.9b00843 31335133

[bb7] Kershaw Cook, L. J., Mohammed, R., Sherborne, G., Roberts, T. D., Alvarez, S. & Halcrow, M. A. (2015). *Coord. Chem. Rev.* **289–290**, 2–12.

[bb8] Piñeiro-López, L., Valverde-Muñoz, F. J., Seredyuk, M., Bartual-Murgui, C., Muñoz, M. C. & Real, J. A. (2018). *Eur. J. Inorg. Chem.* pp. 289–296. 10.1021/acs.inorgchem.7b02272 29048915

[bb9] Rigaku OD (2024). *CrysAlis PRO* . Rigaku Oxford Diffraction, Yarnton, England.

[bb10] Senthil Kumar, K., Šalitroš, I., Heinrich, B., Fuhr, O. & Ruben, M. (2015). *J. Mater. Chem. C.* **3**, 11635–11644.

[bb11] Seredyuk, M., Gaspar, A. B., Ksenofontov, V., Reiman, S., Galyametdinov, Y., Haase, W., Rentschler, E. & Gütlich, P. (2006). *Hyperfine Interact.* **166**, 385–390.

[bb12] Seredyuk, M., Gaspar, A. B., Kusz, J., Bednarek, G. & Gütlich, P. (2007*a*). *J. Appl. Cryst.* **40**, 1135–1145.

[bb13] Seredyuk, M., Haukka, M., Fritsky, I. O., Kozłowski, H., Krämer, R., Pavlenko, V. A. & Gütlich, P. (2007*b*). *Dalton Trans.* pp. 3183–3194. 10.1039/b702574b 17637993

[bb14] Seredyuk, M., Li, R., Znovjyak, K., Zhang, Z., Valverde–Muñoz, F. J., Li, B., Muñoz, M. C., Li, Q., Liu, B., Levchenko, G. & Real, J. A. (2024*a*). *Adv. Funct. Mater.* **34**, 2315487.

[bb15] Seredyuk, M., Znovjyak, K., Valverde-Muñoz, F. J., da Silva, I., Muñoz, M. C., Moroz, Y. S. & Real, J. A. (2022). *J. Am. Chem. Soc.* **144**, 14297–14309. 10.1021/jacs.2c05417 PMC9380689 35900921

[bb16] Seredyuk, M., Znovjyak, K., Valverde-Muñoz, F. J., Muñoz, M. C., Fritsky, I. O. & Real, J. A. (2024*b*). *Dalton Trans.* **53**, 8041–8049. 10.1039/d4dt00368c 38652019

[bb17] Seredyuk, M., Znovjyak, K. O., Kusz, J., Nowak, M., Muñoz, M. C. & Real, J. A. (2014). *Dalton Trans.* **43**, 16387–16394. 10.1039/c4dt01885k 25247462

[bb18] Sheldrick, G. M. (2015*a*). *Acta Cryst.* A**71**, 3–8.

[bb19] Sheldrick, G. M. (2015*b*). *Acta Cryst.* C**71**, 3–8.

[bb20] Shiga, T., Saiki, R., Akiyama, L., Kumai, R., Natke, D., Renz, F., Cameron, J. M., Newton, G. N. & Oshio, H. (2019). *Angew. Chem. Int. Ed.* **58**, 5658–5662. 10.1002/anie.201900909 30753754

[bb21] Spackman, P. R., Turner, M. J., McKinnon, J. J., Wolff, S. K., Grimwood, D. J., Jayatilaka, D. & Spackman, M. A. (2021). *J. Appl. Cryst.* **54**, 1006–1011. 10.1107/S1600576721002910 PMC8202033 34188619

[bb22] Suryadevara, N., Mizuno, A., Spieker, L., Salamon, S., Sleziona, S., Maas, A., Pollmann, E., Heinrich, B., Schleberger, M., Wende, H., Kuppusamy, S. K. & Ruben, M. (2022). *Chem. A Eur. J.* **28**, e202103853. 10.1002/chem.202103853 PMC9305185 34939670

[bb23] Wei, S. Y., Wang, J. L., Zhang, C. S., Xu, X.-T., Zhang, X. X., Wang, J. X. & Xing, Y.-H. (2015). *ChemPlusChem* **80**, 549-558. 10.1002/cplu.201402255 31973413

[bb24] Xing, N., Xu, L. T., Liu, X., Wu, Q., Ma, X. T. & Xing, Y. H. (2014). *ChemPlusChem* **79**, 1198-1207.

[bb25] Yuan, L.-Z., Ge, Q., Zhao, X.-F., Ouyang, Y., Li, S.-H., Xie, C.-Z. & Xu, J.-Y. (2014). *Synth. React. Inorg. Met.-Org. Nano-Met. Chem.* **44**, 1175–1182.

